# Modelling the mass consumption potential of Plant Based Meat: Evidence from an emerging economy

**DOI:** 10.1016/j.heliyon.2024.e24273

**Published:** 2024-01-08

**Authors:** Marvello Yang, Mohammad Nurul Hassan Reza, Qing Yang, Abdullah Al Mamun, Naeem Hayat

**Affiliations:** a Institute of Technology and Business Sabda Setia, Kota Pontianak, Kalimantan Barat, 78121, Indonesia; b UKM - Graduate School of Business, Universiti Kebangsaan Malaysia, 43600, UKM, Bangi, Selangor Darul Ehsan, Malaysia; c Global Entrepreneurship Research and Innovation Centre, Universiti Malaysia Kelantan, Pengkalan Chepa, 16100, Kota Bharu, Kelantan, Malaysia

**Keywords:** Plant-based meat, Consumption value, Intention, Actual consumption

## Abstract

The rising demand for plant-based meat (PBM) has the potential to minimize environmental degradation and save the lives of numerous animals. Furthermore, the intention to consume eco-friendly products triggers people to consume PBM. However, it is essential to understand the factors that influence consumer intentions and actual PBM consumption to enhance its adoption. By incorporating the Theory of Consumption Value, this study examines the impact of health consciousness, health motivation, personal innovativeness, perceived critical mass, perceived cost, and perceived product value on the intention to consume PBM. The impact of intention to consume PBM on actual consumption is also analyzed. Furthermore, the mediating role of the intention to consume PBM in the relationship between these factors and actual consumption behavior is investigated. Using a cross-sectional research design, 978 responses were obtained from Indonesia. The data were analyzed using partial least squares structural equation modelling (PLS-SEM). The results showed that health motivation, perceived critical mass perceived cost, and perceived product value were significant predictors of the intention to consume PBM. However, health motivation and personal innovation had insignificant effects on the intention to consume PBM. Moreover, the intention to consume PBM translated into actual consumption behavior. Furthermore, the intention to consume PBM significantly mediated the relationship between actual consumption behavior and factors other than health motivation, personal innovation, and perceived product value. The findings offer valuable insights for industry, policymakers, and producers interested in PBMA markets in developing focused marketing strategies, improving consumer perceptions, and addressing barriers, such as perceived costs to promote PBM consumption, particularly in emerging markets. Integrating the theory of consumption value and PLS-SEM provides a comprehensive understanding of the underlying dynamics and sheds light on the unique factors driving PBM consumption behavior.

## 1 Introduction

Global meat consumption has significantly increased over the past two decades [1]. The main factors contributing to the increase in meat consumption are the need for protein, energy, and body growth [2,3]. Approximately 230 tons of meat was consumed in 2018, which is expected to increase by approximately 15 % by 2027 [4]. Consequently, the massive demand for meat has caused a natural imbalance among human well-being, animal life, and the planet. In particular, the excessive consumption of animal protein can lead to substantial challenges. Livestock abundance can cause water stress, exacerbate climate change, disrupt natural cycles, and negatively affect nitrogen cycling and other forms of biodiversity [5].

Therefore, a shift toward reducing meat consumption is required in developing countries by replacing it with plant-based meat (PBM). The term “PBM” refers to products that closely match the flavor, texture, and look of traditional meat but are created exclusively from plant-based components [6]. PBM products are often defined as meat alternatives or meat substitutes. These food items are typically made from vegetarian or vegan ingredients and are consumed as a substitute for traditional meat products [7]. The objective of PBM is to offer a meat-like experience for individuals who seek to decrease their consumption of animal products due to ethical, health, or environmental reasons. Common examples of PBM include burgers, sausages, and chicken nuggets made from ingredients such as soy, pea protein, and mushrooms [8]. PBM has experienced a spike in popularity due to rising consumer interest in sustainable food consumption, animal welfare, and health consciousness [9]. PBM has become a trending topic among academics, delineating various emphases on this subject [10]. Plant-based livestock alternatives benefit both human health and the environment [1,11]. Several studies in developed nations have modeled the potential health benefits of PBM [12]. These benefits include investments in PBM by large food companies [3], fresh meat market demand systems [10], and systems for assessing the sustainability of PBM products [11]. Consumption of PBM can enhance public health, decrease greenhouse gas emissions, and preserve natural resources [7]. In addition [13], highlighted a shift in consumer behavior towards PBM, particularly among younger, educated, and higher-income individuals. This trend, evidenced by a decrease in first-time PBM purchases despite market growth, suggests a potential reduction in the demand for traditional meat, aligning with the idea that PBM could contribute to decrease the destruction of animal life. Similarly [14], estimated that a 10 % decrease in PBM prices or a boost in demand for PBM could result in a 0.15 % decrease in U.S. cattle production.

Promoting PBM in Indonesia is essential for eco-environmental development [15]. According to the 2017 Global Vegetarian Index, the demand for PBM in Indonesia has steadily increased. However, PBM is still in its infancy, and only a few businesses offer these products. In addition, 90 % Indonesians have begun consuming healthier meals to boost their immunity [16]. An Indonesian survey revealed that 86 % of the respondents consumed plant-based milk, 49 % dairy product substitutes, 43 % PBM alternatives, 27 % plant-based condiments, 19 % egg substitutes or vegan “eggs,” and 5 % other products [17]. Moreover, due to the simple access to health information online, the Indonesian consumers have increased health awareness in regard to consuming PBM products.

Thus, researchers have revealed that consumers increasingly prefer PBM over conventional meat owing to its perceived health benefits [18]. The perceived benefits of PBM, in terms of safety, quality, and environmental friendliness, have attracted consumers [7,11]. The factors influencing PBMs' consumption levels vary based on consumer perceptions of the products, even if they are introduced with the same objective of promoting sustainable consumption behavior [19]. However, the existing literature on PBM consumption has several limitations. Researchers have shown that consumer attitudes toward PBM are linked to buying intentions and behavioral preferences (Arwanto et al., 2023). Previous studies have mainly concentrated on the diets’ environmental and health benefits [3,20]. However, there has been little focus on examining the particular factors influencing consumer behavior and perceptions of the value of PBM. Therefore, this area offers an exciting opportunity for research that considers the growing intentions of PBM and actual purchasing decisions.

Consequently, the issue of purchasing behavior has been a controversial and highly debated subject in the field of potential factors affecting the actual consumption of PBM in developing nations. Therefore, there is a need to comprehensively understand the factors that influence consumer attitudes and intentions toward PBM and their actual consumption behaviors in emerging countries [18,20]. Despite the wide variety of studies on PBM consumption [7,10,18], the exact mechanisms influencing consumer intentions and actual behaviors are still unknown. Thus [6,21], suggested investigating the factors and intention dynamics of PBM resulting in actual consumption. Furthermore, while research has been conducted on the factors influencing the intention to consume PBM to some extent, there is a lack of studies examining the mediating effect of intention to consume on the relationship between the influencing factors and actual consumption behavior. Exploring the mechanisms underlying mediation will offer a comprehensive understanding of how these factors influence actual consumption behavior through the formation of intentions. Accordingly, the specific research questions of this study are as follows: (a) How do these factors influence consumers' intention and consumption of PBM? (b) Does consumers’ intention behavior affect their actual consumption of PBM? (c) Does consumer intention behavior mediate the relationship between these factors and the actual consumption of PBM?

By integrating the theory of consumption value and a quantitative approach, this study investigates the factors influencing consumer attitudes and intentions toward PBM in emerging countries such as Indonesia. By examining multiple dimensions, such as health consciousness, health motivation, personal innovativeness, perceived critical mass, perceived cost, and perceived product value, this study enhances the knowledge of consumer behavior in the context of PBM. Additionally, incorporating the intention to consume as a mediator and examining its role in the relationship between the factors and actual consumption behavior is a novel contribution. This approach reveals the underlying mechanisms that influence the actual PBM consumption behavior and allows for a more sophisticated understanding of the decision-making process.

The remainder of this paper is organized as follows. The literature review discusses the theory of consumption values and hypotheses development. The methodology section provides an overview of the data collection and analysis processes. The findings of this study are presented in the discussion section. The theoretical and practical implications, along with the limitations of this study, are presented in the implications section. Finally, the concluding remarks are presented.

## 2 Literature review

### 2.1 The theory of consumption value

The consumption value theory, developed by Ref. [22] emphasizes consumer awareness of how consumption affects the environment. This theory is consistent with decade-long efforts to comprehend socially and environmentally responsible consumption [23]. The existing research concentrates on the responses to environmentally friendly products [24] or the consequences of various situational factors, making it difficult to determine how and why environmentally responsible consumer behavior varies among individuals [25]. Previous research on socially responsible consumption has explored individuals’ consumption behaviors [23,26]. Thus [24], suggested that changes in consumer behavior, such as substituting sustainably produced meat with green products, can reduce the environmental destruction and health issues caused by animal meat production and consumption.

[22] stated that social, emotional, epistemic, and conditional values are the dominant factors in the consumption value theory. Social value refers to demographic stereotypes in which others' opinions can influence consumer behavior and decisions. According to Ref. [27]; consumers' social pressures influence their product consumption. Emotional value is the consumer's perceived utility derived from the product consumed [22]. Consumers gain knowledge regarding the value of the consumed product, which is known as epistemic value. Finally, conditional value provides the perceived utility of the consumed product to change decision making [26]. Different factors affecting individuals' consumption values, such as health awareness, health motivation, and personal innovativeness, are new concepts in the consumption value theory. Perceived critical mass can be understood as a social value through which a consumer can influence the other consumers who will purchase a new product. Perceived cost and product value are defined as emotional and epistemic values, respectively. Intention is the conditional value of perceived use that may change consumers' decisions to consume green products. Researchers have recommended context-specific studies to explore these factors and understand consumer behavior.

### 2.2 Hypotheses development

Based on the consumption value theory, many studies have been conducted on individual choice behavior in developed countries. However, the results cannot be generalized to target markets in developing countries, such as Indonesia [28,29]. This study provides new theoretical insights into green product consumption behavior, supported by empirical findings. This study broadens our understanding of consumption intention as a mediating variable in the relationship between these factors and green behavior. This implies that intention is an important factor in promoting green products in developing markets. Little attention has been paid to the consumption value theory, emphasizing the PBM in sustainability studies. For example [30], utilized theory of consumption value (TCV) to explain the adoption of food delivery systems, address ethical consumers [29], and understand the sustainable consumption of organic food [23].

This study justifies the integration of TCV for several reasons. The PBM choice manifests consumers’ green behavior when faced with a choice. The TCV is commonly used to study consumer behavior in green product consumption [25,31]. Moreover, TCV offers a valuable technique for understanding consumer behavior as it incorporates a well-known view in consumer behavior research. Furthermore, TCV has been successfully extended to the green context to gain insight into the consumer value of green products [32]. Based on these observations, we used TCV to investigate consumer behavior in green product consumption, such as PBM, which is still evolving. Consequently, this study integrates factors such as health awareness, health motivation, personal innovativeness, perceived critical mass, perceived cost, and perceived product value. It examines their impact on consumption intentions, leading to actual consumption. The following sub-sections demonstrate the rationale for hypothesis building.

#### 2.2.1 Health consciousness

Health consciousness refers to people's awareness of and concern for their health and well-being. Health-conscious people are likely to consider PBM favorably because of its perceived positive effects on health. The consumption value theory proposes that people make consumption decisions according to their values, interests, and aims [11]. stated that an individual's consumption behavior can be altered to reduce the consumption of animal meat and improve health [7]. revealed that choosing PBM over traditional products can reduce environmental degradation. Increasing consumer consciousness, owing to technological advancements, has changed the way people consume technology-based products [13]. This change can benefit the environment and human health, and enhance the quality of life [33]. Health-conscious people may view PBM as a healthier option than traditional meat and are more likely to consume it. In this regard [34], found that health consciousness was positively associated with the demand for PBM. Additionally [35], revealed that the perception of healthiness or consciousness significantly impacted the level of purchase intent for PBM. Based on this explanation, this study proposes the following hypothesis.

H1 Health consciousness has a significant effect on the intention to consume PBM.

#### 2.2.2 Health motivation

An important factor that peoples consider when choosing PBM products is health motivation [2]. Personal importance or desire associated with preserving or enhancing an individual's health is defined as health motivation [28]. stated that individuals' motivation to consume technology-based products, such as PBM, is related to their emotional state. The emotional motivation to consume such products increases because of their advantages over conventional products [36]. Thus, the positive attitudes and intentions expressed by individuals motivated by health will translate into actual behavior. People who prioritize their health are more likely to adhere to their intentions and adopt PBM as part of their dietary options. Accordingly, this study proposes the following hypothesis.

H2 Health motivation has a significant effect on the intention to consume PBM.

#### 2.2.3 Personal innovativeness

[37] defined individual innovativeness as the willingness to experiment with new information technologies. Individual innovativeness reflects an individual's primary characteristics when exposed to innovation [38]. Innovativeness has been studied extensively as a factor in innovation adoption behavior. In line with this [39], stated that consumers with higher levels of personal innovativeness are likely to develop more favorable attitudes toward technology-based products, new foods, or dietary options when they are younger. These individuals may view PBM as a novel and exciting alternative to conventional meat and are encouraged by the excitement of exploring new flavors and experiences. They are more likely to consume PBM frequently because of their openness to innovation and readiness to try new things. According to Ref. [40]; there exists a strong correlation between consumer preferences within the PBM market, underscoring the significance of promoting a multitude of plant-based alternatives to cater to the innovative preferences of consumers. However, individual differences in innovativeness and the propensity to adopt innovation influence how people respond to new ideas, practices, and objects [41]. Consequently, this study proposes the following hypothesis.

H3 Personal innovativeness has a significant effect on the intention to consume PBM.

#### 2.2.4 Perceived critical mass

Critical mass is a subset of a population that voluntarily contributes significantly to a collective action [39]. [42] found that critical mass is strongly associated with peer interactions in group software adoption. In general, the theory of critical mass is essential for explaining the adoption and use of interactive communication media because the value of the network increases as the number of media users increase [38]. Perceived critical mass assesses individuals' perceptions of their peers' technology usage. It measures individuals' perceptions of their peers’ technology use, which can be influenced by exposure rather than direct observations [43]. In the context of PBM consumption, the perceived critical mass suggests that individuals who perceive many people as consuming PBM are more likely to develop an intention to consume it. By creating a sense of social norms, the concept of perceived critical mass may make PBM more appealing and acceptable. Based on the above justifications, this study proposes the following hypothesis.

H4 Perceived critical mass has a significant effect on the intention to consume PBM.

#### 2.2.5 Perceived cost

Perceived cost defines the perception of expenditures related to consuming PBM, including the cost of the products, any additional expenses for meal planning or preparation, and any perceived tradeoffs regarding flavor or convenience compared to traditional meat. Thus, individuals’ paying behavior is influenced by their willingness to pay and social expectations [44]. Prior studies have identified perceived cost as the most influential factor in adoption [45]. The perceived cost refers to the financial burden associated with the acquisition, use, and maintenance [46]. Thus, it is predicted that people who believe PBM to be more expensive may be reluctant to express their intention to consume it. Higher perceived costs might create a perceived barrier that decreases the intention to engage in the behavior of consuming PBM [47]. conducted research into the willingness to switch to meat substitutes and found that a considerable proportion of traditional meat consumers are price-sensitive. As such, reducing the cost of PBM products may be a crucial factor in encouraging their consumption. Based on the preceding discourse, the following hypothesis is proposed.

H5 Perceived cost has a significant effect on the intention to consume PBM.

#### 2.2.6 Perceived product value

There is substantial support for the impact of perceived value on environment-related behaviors, such as the consumption of green products [32]. The perceived value theory also supports the idea that buying eco-friendly products can influence people's behavior when making environmental decisions [22]. The consumption value theory reveals that various consumption values can influence consumer behavior. These include functional, social, emotional, and conditional values [10]. Values associated with green products are the most influential factors that are considered while purchasing [46]. The perceived advantages, qualities, and attractiveness of PBMs are all included in perceived product value, which people consider when assessing and choosing foods to consume. People who believe that PBM is more valuable than traditional meat tend to consume it. Thus, positive product value perceptions promote a positive attitude toward PBM and increase the likelihood of both intention and actual consumption behaviors. Based on the above discussion, this study proposes the following hypothesis.

H6 Perceived product value has a significant effect on the intention to consume PBM.

#### 2.2.7 Intention to consume

Consumers' product selections are influenced by their purchase intentions [48]. Researchers have shown that individuals’ purchase intentions significantly influence their green product consumption decisions (Qi and Ploeger, 2020). Individuals make decisions based on ideas, attitudes, and values. These intentions act as motivating factors that direct and forecast consequent behavior. Given the increase in organic food consumption, purchasing intentions have been optimistic [49]. Therefore, when individuals understand the importance and advantages of PBM, such as its advantages in terms of health, sustainability, and ethics, they may adopt a positive attitude and intention to include it in their dietary options. This indicates that purchasing intentions substantially affect the consumption of organic products such as PBM [44]. Based on the above explanation, this study proposes the following hypothesis.

H7 Intention to consume PBM has a significant effect on actual consumption.

#### 2.2.8 Mediating effect of intention to consume

Purchase intention significantly predicts consumer decisions to consume environment-friendly products. To effectively respond to the expansion of the green food market, it is necessary to examine consumer purchasing behavior, with particular emphasis on purchase intention [50]. Several studies have emphasized the relationship between consumer attitudes, purchase intentions, and behavior [48]. [51] discovered that health concerns, environmental concerns, knowledge and awareness, and price are the most influential factors in consumers’ organic food-purchasing behavior. However, this study proposes that the influence of these factors on the actual consumption of PBM is mediated by the intention to consume. In other words, the impact of health consciousness, health motivation, personal innovativeness, perceived critical mass, perceived cost, and perceived product value on actual consumption is indirect, and occurs through their influence on the intention to consume PBM. It is anticipated that individuals with a stronger intention to consume PBM will be more likely to translate their intentions into actual behavior by consuming PBM. Based on the above discussion, this study proposes the following hypothesis.

H8 Intention to consume PBM significantly mediates the relationship between health consciousness, health motivation, personal innovativeness, perceived critical mass, perceived cost, perceived product value, and actual consumption of PBM.

**Fig. 1:**
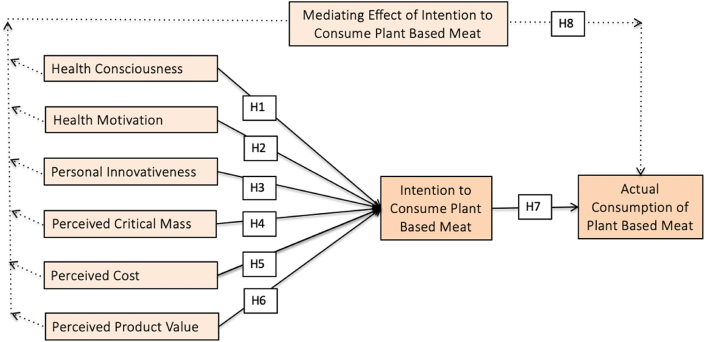
Research framework.

All the relationships hypothesized above are presented in Fig. 1.

## 3 Methodology

### 3.1 Research design

This study used a quantitative approach. A questionnaire-based survey was conducted to obtain detailed information on the health consciousness, health motivation, personal innovativeness, perceived critical mass, perceived cost, perceived product value, intention to consume, and actual consumption of PBM. The research ethics committee of Institute of Technology and Business Sabda Setia, Indonesia have approved this study (Approval Number: 295AA/ITBSS/I/2023). This study has been performed in accordance with the Declaration of Helsinki. Written informed consent for participation was obtained from respondents who participated in the survey.

The geographical context of this study was Indonesia. The study population included Indonesian consumers, and the unit of analysis was the individuals who consumed PBM. Particularly, the respondents were selected based on their history of PBM consumption in the past six months. They were categorized according to their consumption habits, with regular consumers indicating frequent consumption (at least once a week) and occasional consumers reporting infrequent consumption (less than once a week). However, PBM is a relatively new research topic in the Indonesian food industry. Therefore, convenience sampling was applied owing of geographical proximity, respondents’ availability at a given time, and willingness to participate in this study [52]. The questionnaire used in this study was translated into Indonesian by an expert. A pilot test was conducted by collecting data from 100 respondents to reduce ambiguity and ensure that the questionnaire and sampling techniques were well-understood, thereby ensuring content validity. Further, to determine the approximate sample size, a G*Power test was conducted using an effect size (f^2^) of 0.15, an alpha error probability of 0.05, a power (1-beta error probability) of 0.95, and seven predictors. The initial estimated sample size calculated by G*Power was 153, but we decided to increase the sample size to 1500 to avoid the challenges of a small sample size.

The questionnaire was distributed through digital platforms such as email, massage, Facebook posts, and WhatsApp, with a request to share the Google form link on their social media pages and with their contacts. The survey was conducted from January 15, 2023, to February 25, 2023. The participants received two reminders, the most common number used in participant email surveys [53]. Each participant received a personalized link to the survey. This allowed them to access the questionnaire without providing a user name or password, which had a positive effect on the response rates [54]. The reminder was delivered the following week. The researchers sent an additional reminder to non-respondents between 9 and 17 days after the second reminder. Academic literature indicates that sending a reminder one week rather than one month after the invitation is more effective in increasing the number of participants who have already completed the questionnaire [55]. Those who completed the questionnaire did not receive reminders. Complete data were collected from 978 respondents.

### 3.2 Survey Instrument

The measurement items for health consciousness, health motivation, personal innovativeness, perceived critical mass, perceived cost, and perceived product value were adapted from Refs. [[56], [57], [58], [59], [60]]; respectively. Each construct includes five items. The intention to consume PBM was measured using five items adapted from Refs. [61,62]. The actual consumption of PBM was measured using a single item, that is, how frequently plant-based meat was consumed, adapted from Ref. [62]. This study used a five-point Likert scale (“strongly disagree,” “disagree,” “neither agree” nor “disagree,” “agree,” and “highly agree”) to identify the responses of the participants. A Likert scale is simple to construct and likely to produce high validity and reliability [63]. In addition, from the participants’ perspective, it was easy to read and complete the Likert-scale-based questionnaire. A five-point Likert scale is the most widely used measurement scale for questionnaire-based surveys. All items used in this study presented as Supplementary Material 1 Table S1. Survey Instrument.

### 3.3 Data analysis method

The researchers used partial least squares structural equation modelling (PLS-SEM) using Smart-PLS, Version 3.1 to analyze the data and validate the proposed model. The SEM-PLS is recommended for exploratory research to develop new theories or extend the established theories [64]. This method is appropriate for analyzing the causal relationships among the constructs in a complex model that includes many constructs and indicators [65]. Moreover, PLS-SEM is suitable for testing research models from a predictive perspective [66,67]. The current exploratory study aims to extend the consumption value theory, emphasizing a novel research area: the translation of the intention to consume PBM to actual consumption behavior. The proposed research model includes various dimensions (factors) of green-product consumption behavior to investigate the causal relationships between the constructs based on the prediction perspective. Considering the aforementioned advantages, this study employed SEM-PLS for data analysis.

## 4 Findings

### 4.1 Demographic characteristics

Table 1 shows the demographic information. Most participants in this study were women, 54.4 % of the total. The age group that most commonly consumed PBM was 18–25 years accounting for 65 % of participants. A large majority of participants were full-time employees with 54.8 % of total. The highest academic attainment held by the majority of the participants was a high school diploma, at 43.3 %. Moreover, approximately 42.6 % of the participants had a monthly income below Rp. 3 million.

**Table 1:** Demographic characteristics.

	N	%			N	%
*Gender*				*Education*		
Male	446	45.6		No High School Diploma	177	18.1
Female	532	54.4		High School Diploma	423	43.3
Total	978	100.0		Bachelor Degree	228	23.3
				Master Degree	107	10.9
*Age Group*				Doctoral Degree	43	4.4
18–25 years old	636	65.0		Total	978	100.0
26–30 years old	86	8.8				
31–35 years old	111	11.3		*Monthly Income*		
36–40 years old	90	9.2		< Rp. 3 million	417	42.6
above 40 years old	55	5.6		≥ Rp. 3 to Rp. 5 million	248	25.4
Total	978	100.0		≥ Rp. 5 to Rp. 7 million	111	11.3
				≥ Rp. 7 to Rp. 9 million	48	4.9
*Employment Status*				above Rp. 9 million	154	15.7
Employed (Full-time)	536	54.8		Total	978	100.0
Employed (Part-time)	232	23.7				
Self-Employed	127	13.0				
Student	38	3.9				
Unemployed/Retired	45	4.6				
Total	978	100.0				

**Note:** Indonesian Rupiah (Rp); 1 USD = 15151.45 Rp.

### 4.2 Preliminary analysis

The data were cross-sectional and collected from a single source; therefore, multivariate normality and common method bias were examined to ensure construct validity.

#### 4.2.1 Common method bias

Common method bias (CMB) occurs in surveys when researchers collect data using the same method, potentially resulting in artificial inflation of relationships [68]. Herman's single-factor test was used to assess the CMB. The results showed that the variance explained by a single factor was 34.32 %, which was lower than 50 %, indicating that CMB was not an issue in this study [69]. suggested that CMB can be predicted using the full collinearity of the constructs. The results presented in Table 2 show that all variance inflation factor (VIF) values were less than five, demonstrating no involvement of CMB [69].

**Table 2:** Full collinearity test.

	HCS	HMO	PIN	PCM	PCT	PPV	ICP	ACO
Variance Inflation Factors	2.193	1.682	2.518	2.428	2.349	1.727	1.932	1.037

**Note:** HCS - Health Consciousness; HMO - Health Motivation; PIN - Personal Innovativeness; PCM - Perceived Critical Mass; PCT - Perceived Cost; PPV - Perceived Product Value, ICP - Intention to Consume Plant Based Meat; ACO - Actual Consumption of Plant Based Meat.

#### 4.2.2 Multivariate normality

According to the recommendations of [70]; this study examined the multivariate normality by determining the *p*-value of Mardia's skewness and kurtosis. The Web Power of this online calculator was used to obtain the results. The results showed that the p-values for skewness and kurtosis were lower than 0.05, indicating significance and deviation from the assumption of multivariate normality of the data.

### 4.3 Validity and reliability

We assessed the measurement and structural models to validate the proposed framework. The measurement model was evaluated by examining the reliability and validity of the constructs, whereas the structural model was evaluated by assessing the interrelationships between the constructs. The reliability of the constructs was verified by examining Cronbach's alpha and composite reliability. Validity was analyzed by assessing the convergent and discriminant validity. Convergent validity was established by checking the average variance extracted (AVE), whereas discriminant validity was checked using the Fornell and Larcker criterion, cross-loadings, and the heterotrait-monotrait (HTMT) ratio.

According to the recommendation of [65]; Cronbach's alpha and composite reliability values should be greater than 0.70. The results presented in Table 3 show that the Cronbach's alpha and composite reliability values were higher than 0.70, indicating adequate reliability [65]. Convergent validity suggests that a set of indicators represent the same underlying construct, as evidenced by its unidimensionality, as checked by the AVE, and the value should be greater than 0.50. The results shown in Table 3 demonstrate that the AVE values were higher than 0.50, indicating sufficient convergent validity of the constructs [71]. Furthermore, the VIF value of each construct, which should be less than five, was assessed. The results presented in Table 3 show that all values were lower than five, indicating no multicollinearity issues.

**Table 3:** Reliability and validity.

Variables	No. Items	Mean	Standard Deviation	Cronbach's Alpha	Composite reliability (rho_a)	Composite reliability (rho_c)	Average Variance Extracted	Variance Inflation Factors
HCS	5	4.435	0.549	0.813	0.845	0.870	0.578	2.332
HMO	5	4.339	0.535	0.765	0.778	0.837	0.510	1.837
PIN	5	4.235	0.632	0.843	0.852	0.889	0.619	2.563
PCM	5	4.065	0.735	0.843	0.849	0.888	0.614	2.655
PCT	3	4.089	0.591	0.777	0.782	0.871	0.692	2.006
PPV	5	4.224	0.578	0.824	0.842	0.877	0.591	1.726
ICP	5	4.097	0.573	0.783	0.823	0.852	0.541	1.000
AOC	1	3.971	0.806	–	–	–	–	–

**Note:** HCS - Health Consciousness; HMO - Health Motivation; PIN - Personal Innovativeness; PCM - Perceived Critical Mass; PCT - Perceived Cost; PPV - Perceived Product Value, ICP - Intention to Consume Plant Based Meat; ACO - Actual Consumption of Plant Based Meat.

**Fig. 2:**
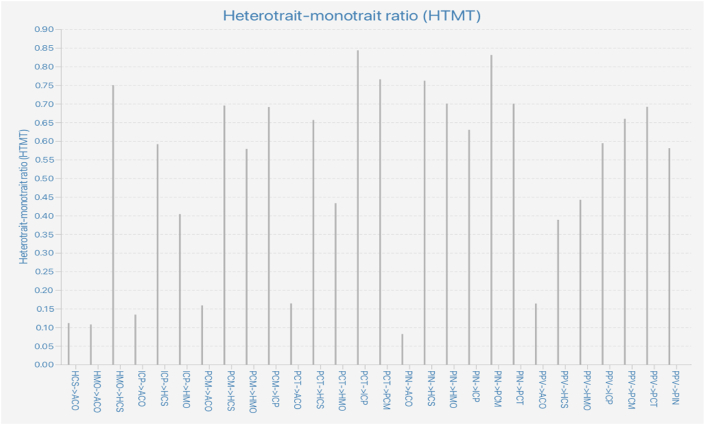
Heterotrait-Monotrait ratio (HTMT) Matrix.

The discriminant validity of the constructs was analyzed by assessing the HTMT, cross-loadings, and Fornell-Larcker criterion [72]. Regarding the HTMT ratio, the suggested threshold is lower than 0.90 [65]. Fig. 2 shows that the HTMT values were lower than 0.90, demonstrating adequate discriminant validity. Additionally, the cross-loadings were sufficient to support discriminant validity. The results are presented in Supplementary Material 2 Table S2. Loading, Cross-Loading, and Fornell–Larcker Criterion. According to the result, the loadings of the intended construct are higher than their cross-loadings on the other constructs. Furthermore, the results of the Fornell–Larcker criteria were evaluated by comparing the square root of the AVE with the correlation coefficients. The results presented in Supplementary Material 2 Table S2. Loading, Cross-Loading, and Fornell–Larcker Criterion show that the square root of the AVE is larger than the correlation coefficients between the construct and the other constructs [65], suggesting adequate discriminant validity.

### 4.4 Path analysis

**Fig. 3:**
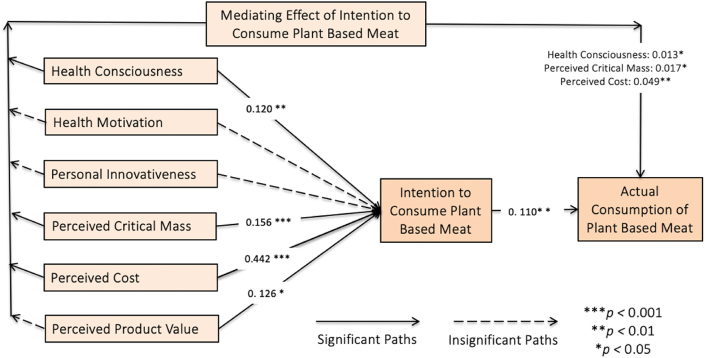
Final model with outputs.

The results of the path analysis are presented in Table 4 and Fig. 3. According to the results, the positive coefficient value for the effect of health consciousness on the intention to consume PBM (H1) with a significant *p*-value of 0.007 (*p* < 0.05) confirmed a significant and positive effect of health consciousness on the intention to consume PBM, supporting H1. However, the negative coefficient value for the effect of health motivation on the intention to consume PBM (H2) with an insignificant *p*-value of 0.253 (*p* > 0.05); showed a negative and insignificant effect of health motivation on the intention to consume PBM, rejecting H2. The coefficient value for the effect of personal innovativeness on the intention to consume PBM (H3) was positive with an insignificant *p*-value of 0.250 (*p* > 0.05); demonstrating an insignificant positive effect of personal innovativeness on the intention to consume PBM. Hence, H3 was not supported. On the other hand, the positive coefficient value for the effect of perceived critical mass on the intention to consume PBM (H4) with a significant *p*-value of 0.000 (*p* < 0.05); confirming a significant and positive effect of perceived critical mass on the intention to consume PBM. Therefore, H4 is supported. Moreover, the positive coefficient value for the effect of perceived cost on the intention to consume PBM (H5) a significant *p*-value of 0.000 (*p* < 0.05); indicating a significant and positive effect of perceived cost on the intention to consume PBM. Hence, H5 is supported. Additionally, the positive coefficient value for the effect of perceived product value on the intention to consume PBM (H6) with a significant *p*-value of 0.022 (*p* < 0.05); demonstrating a significant and positive effect of perceived product value on the intention to consume PBM. Therefore, H6 is also supported. Finally, the positive coefficient value for the effect of the intention to consume PBM on actual consumption (H7) with a significant *p*-value of 0.003 (*p* < 0.05); indicating a significant and positive effect of the intention to consume PBM on actual consumption. Thus, H7 is supported.

**Table 4:** Hypothesis testing.

Hypothesis		Beta	CI Min	CI Max	*t* Value	*p* Value	*r*^*2*^	Supported?
H1	HCS → ICP	0.120	0.038	0.200	2.441	0.007	0.527	Yes
H2	HMO → ICP	−0.034	−0.113	0.056	0.665	0.253		No
H3	PIN → ICP	0.041	−0.069	0.135	0.673	0.250		No
H4	PCM → ICP	0.156	0.085	0.231	3.517	0.000		Yes
H5	PCT → ICP	0.442	0.341	0.538	7.326	0.000		Yes
H6	PPV → ICP	0.126	0.029	0.233	2.016	0.022		Yes
H_7_	ICP → ACO	0.110	0.045	0.176	2.792	0.003	0.012	Yes
*Mediating Effect*								
H8_a_	HCS → ICP → ACO	0.013	0.003	0.024	2.027	0.021		Yes
H8_b_	HMO → ICP → ACO	−0.004	−0.013	0.007	0.608	0.272		No
H8_c_	PIN → ICP → ACO	0.005	−0.007	0.018	0.609	0.271		No
H8_d_	PCM → ICP → ACO	0.017	0.006	0.033	2.104	0.018		Yes
H8_e_	PCT → ICP → ACO	0.049	0.020	0.079	2.730	0.003		Yes
H8_f_	PPV → ICP → ACO	0.014	0.002	0.034	1.363	0.086		No

**Note:** HCS - Health Consciousness; HMO - Health Motivation; PIN - Personal Innovativeness; PCM - Perceived Critical Mass; PCT - Perceived Cost; PPV - Perceived Product Value, ICP - Intention to Consume Plant-Based Meat; ACO - Actual Consumption of Plant-Based Meat.

### 4.5 Mediating effect

The mediating effects are presented in Table 4. According to the results, the positive coefficient value for the mediating effect of the intention to consume PBM between the relationship of health consciousness and actual consumption of PBM (H8_a_) with a significant *p*-value of 0.021 (*p* < 0.05) showing a significant and positive mediating effect, thus supporting H8_a_. However, the negative coefficient value for the mediating effect of the intention to consume PBM on the link between health motivation and actual consumption of PBM (H8_b_) with an insignificant *p*-value of 0.272 (*p* > 0.05); indicating an insignificant mediating effect, thus rejecting H8_b_. The coefficient value of the mediating effect of the intention to consume PBM on the link of personal innovativeness and actual consumption to PBM (H8_c_) was positive with an insignificant *p*-value of 0.271 (*p* > 0.05); suggesting an insignificant mediating effect, thus rejecting H8_c_. However, the positive coefficient value for the mediating effect of the intention to consume PBM on the link between perceived critical mass and actual consumption of PBM (H8_d_) with a significant *p*-value of 0.018 (*p* < 0.05); indicating a significant mediating effect, thus supporting H8_d_. The positive coefficient value for the mediating effect of the intention to consume PBM on the link between perceived cost and actual consumption (H8_e_) with a significant *p*-value of 0.003 (*p* < 0.05); showing a significant mediating effect, thus supporting H8_e_. Finally, the coefficient value for the mediating effect of the intention to consume PBM between the link of perceived product value and actual consumption to PBM (H8_f_) was positive with an insignificant *p*-value of 0.086 (*p* > 0.05); indicating an insignificant mediating effect, thus rejecting H8_f_.

## 5 Discussion

Given the deteriorating environmental problems, there is a growing consumer demand for environmentally responsible products. Based on the findings in Table 4, health consciousness directly influences the intention to consume PBM (H1). In other words, most consumers recognized the benefits of substituting meat with PBM. Additionally, the relationship between health awareness and actual consumption of PBM was mediated by purchase intention (H8_a_). These findings indicate that health awareness and the intention to preserve natural systems develop health consciousness and the intention to consume PBM, which translates into actual consumption. This result is consistent with the findings of previous studies [33,73,74]. Consumers who are conscious of their health and environmental degradation may choose PBM as a preventive strategy against diseases that may have long-term effects on their future well-being.

On the contrary, health motivation (H2) and personal innovation (H3) had insignificant effects on the intention to consume PBM. In contrast to Ref. [28]; most studies indicate that healthy living motivation and personal innovativeness are necessary to encourage consumers to consume PBM [20,62]. Personal innovation in technology (i.e., a person's propensity to experiment with new technologies) appeared to be insignificant in explaining intention. These findings are also inconsistent with those of previous studies [75,76]. There are several possible reasons for these contradictory results. Individuals with low levels of personal innovativeness tend to be reluctant to experiment with, and fear embracing, new technologies [77]. found that innovativeness is a weak predictor of an individual's behavior. In this study, consumers with low innovativeness were less able to persuade others to test new products for a healthy lifestyle and a healthier natural environment. Moreover, the mediating effects of the intention to consume PBM on the relationships of health motivation (H8_b_), personal innovation (H8_c_), perceived product value (H8_f_), and actual consumption of PBM were insignificant.

In addition, the relationship between perceived critical mass and the intention to consume PBM was supported (H4), indicating that consumers consider the information they receive from references (parents and close acquaintances) crucial for encouraging their intention. Potential environmental hazards caused by inconsequential consumer activities can be mitigated if reasonable and provocative messages are conveyed to alter consumer mindsets. Moreover, perceived cost (H5) and perceived product value (H6) significantly influenced the intention to consume PBM. These results indicate that when consumers comprehend the value and cost of PBM products, they increase their consumption, thereby contributing to environmental development. Thus, enhancing a product's perceived value amplifies the benefits of PBM for consumers [36]. Educating consumers on environmental sustainability is essential for spending money on green products. Further, the findings demonstrate a significant effect of the intention to consume PBM on actual consumption (H7), indicating that the intention to consume PBM is a determinant of actual consumption behavior. In addition, the mediating role of the intention to consume PBM significantly influenced the relationship between perceived critical mass and actual consumption (H8_d_). This result is supported by Ref. [43]; who found that perceived critical mass significantly contributes to consumers' decisions to purchase goods. Moreover, this study produced a significant mediating effect on the link between perceived cost and actual consumption of PBM (H8_e_). This result is in line with the previous study by Ref. [45]; who found that consumers who perceive the benefits of a product are more inclined to be willing to pay for it.

### 5.1 Theoretical implications

This study contributes to the existing literature in several ways. It adds to the existing body of knowledge by employing the theory of consumption value in the context of PBM consumption. It advances the theoretical understanding of how factors influence the intention to consume PBM, and subsequently, actual consumption behavior. This study also contributes to the theory of consumption value [22] by identifying prospective factors that influence the intention to consume PBM and their effects on individuals’ green consumption decisions. Previous research has focused primarily on the direct relationship between consumption value and individual choice [23,29], whereas this study focuses on the direct and indirect influences of the intention to adopt green consumption in the context of PBM. In this regard, this study demonstrates the importance of considering the role of another value-related construct as a mediating variable in the theory of consumption value and explains the relationship between context-specific consumption values that have not been investigated in previous studies (e. g., Refs. [23,29,30]. Moreover, this study addresses the problem of the paucity of studies by providing empirical evidence of the relationship between the factors and the intention to consume PBM, leading to actual purchases, to explain the purchasing behavior of young, educated consumers regarding PBM. Finally, this study was conducted in Indonesia, which is an emerging country, and thus, contributes to the contextual diversity of the literature on PBM consumption. It acknowledges that cultural, economic, and social factors shape consumer behavior and motivation. Additionally, this study sheds light on the unique factors that influence PBM consumption in emerging markets.

### 5.2 Practical implications

The findings of this study can guide companies and governments in emerging countries in formulating strategies for the development, sale, and consumption of PBM. For instance, to improve customers' intentions to consume and their actual consumption, it may be helpful to emphasize the health advantages of PBM products, target consumers concerned about their diet, and emphasize the worth and accessibility of these products. Additionally, promotions and advertisements containing educational messages about PBM should be publicized on popular platforms such as YouTube, Instagram, and Tik Tok. A lack of information about PBM is likely to reduce a product's marketability and consumer confidence. The significant contribution of cost and product value to intention and behavior suggests that consumers are sensitive to the cost and value of a product. Consumers evaluate the advantages of consuming PBM and consider how their efforts to alter their consumption patterns contribute to environmental conservation. Moreover, strategic positioning of price and product value allows consumers to support environmental sustainability [41]. The availability of these products drives consumer consumption to mitigate global warming. In summary, maximizing product price and value, particularly through ecological certification, can be an option for generating sales.

This study also revealed that consumers are aware of the contribution of PBM consumption in reducing environmental damage. Thus, encouraging consumers to adopt PBM is crucial for addressing climate change issues [15]. Consequently, governments should take initiatives to enhance low-income consumers' perceptions of the environmental value and cost of products, particularly when consuming PBM. Several preventive measures can be implemented in this regard. For instance, issuing fixed fines or penalties can be used as a preventive technique to successfully manage waste concerns [33]. confirmed that enhancing consumers' self-awareness is essential for fostering environmental responsibility. Government notices and promotional campaigns significantly enhance consumers' knowledge regarding purchasing environmentally responsible products. Therefore, policymakers must take decisive action in implementing policies that encourage individuals to develop intentions to engage in environmentally responsible behavior. Green development and corporate social responsibility are essential to the market. Implementing an efficient procurement process can help reduce the manufacturing cost of green products and make them more accessible to low-income groups. Thus, policymakers and practitioners should take initiatives to increase consumers’ intentions to consume PBM and achieve sustainable development.

### 5.3 Limitations and future recommendations

This study adopted a quantitative approach using survey data. Thus, future studies should consider a longitudinal or mixed-method approach to gain a more comprehensive understanding of the causal relationships and temporal dynamics. The geographical context of this study is Indonesia, an emerging country. Although this context offers valuable insights, it may restrict the generalizability of the findings to other geographical or cultural settings. Future research should consider using more diverse samples from various countries to improve the external validity of these findings. Additionally, as this study focuses on mediating variables, other possible factors, such as moderation, should be considered in future studies. Furthermore, researchers have addressed not only consumers’ intentions but also cultural and geographical contexts or government regulations that may influence their behavioral actions in consuming PBM. Therefore, these factors should be included in future studies focusing on PBM consumption. Furthermore, as this study did not consider religious factors, e.g., religious beliefs or perceptions, it is recommended that future investigations delve into the impact of these factors on the decision to consume PBMs. The diverse religious landscape in any geographical context highlights the significance of exploring the intersection of religious values and dietary choices in understanding the factors that influence PBM consumption. Furthermore, to obtain a more comprehensive picture of the demographic influences on PBM consumption, it is suggested that future research consider incorporating additional demographic variables to provide a more comprehensive picture of the demographic influences on PBM consumption.

## 6 Conclusion

In the context of PBM, prior research has paid little attention to investigating the effects of consumption value factors on consumers' intentions translating into actual behavior. This study explored the factors influencing the intention to consume and actual consumption of PBM in Indonesia. The research objectives were achieved by incorporating the theory of consumption value as the theoretical foundation, producing insightful findings for both the theoretical and practical domains. This study examined the impact of key influential factors (i.e., health consciousness, health motivation, personal innovativeness, perceived critical mass, perceived cost, and perceived product value) on the intention towards actual behaviors to consume PBM. The results confirm that personal innovativeness, perceived critical mass, perceived cost, and perceived product value significantly drive consumers' intentions to consume PBM. However, the lack of health motivation and health consciousness among Indonesians restrict their intention to consume PBM. This may be due to consumers’ inadequate willingness and motivation to consume PBM, as this concept is in its early stages in Indonesia. Moreover, the findings revealed that the intention to consume PBM translated into actual consumption. Additionally, the intention to consume PBM mediated the relationships among health consciousness, perceived critical mass, and perceived cost and actual consumption of PBM. However, the mediating impact of intention to consume PBM had insignificant effects on the relationships between health motivation, health consciousness, perceived product costs, and actual consumption. The findings of this study can guide marketing and communication policies, product development, pricing decisions, social influence campaigns, and public health programs. Companies and policymakers should successfully encourage the consumption of PBM by highlighting its health advantages, value, and affordability, eventually resulting in more sustainable dietary choices.

## Ethical approval

The research ethics committee of Institute of Technology and Business Sabda Setia, Indonesia have approved this study (Approval Number: 295AA/ITBSS/I/2023). This study has been performed in accordance with the Declaration of Helsinki.

## Consent to participate

Written informed consent was obtained from respondents who participated in the survey.

## Consent to publish

All authors approved the manuscript and give their consent for submission and publication.

## Funding

This research received no specific grant from any funding agency in the public, commercial, or not-for-profit sectors.

## Availability of data and materials

The original contributions presented in the study are included in the article/Supplementary Material 3, further inquiries can be directed to the corresponding author/s.

## CRediT authorship contribution statement

**Marvello Yang:** Conceptualization, Investigation, Methodology, Writing – original draft. **Mohammad Nurul Hassan Reza:** Conceptualization, Investigation, Methodology, Writing – original draft. **Qing Yang:** Conceptualization, Formal analysis, Methodology, Writing – review & editing. **Abdullah Al Mamun:** Conceptualization, Formal analysis, Methodology, Writing – review & editing. **Naeem Hayat:** Conceptualization, Investigation, Methodology, Writing – original draft.

## Declaration of competing interest

The authors declare the following financial interests/personal relationships which may be considered as potential competing interests:Corresponding author is the associate editor of Heliyon (Business and Management).
