# Supplementary material for: Modelling the mass consumption potential of Plant Based Meat: Evidence from an emerging economy
Source: Heliyon. 2024 Jan 8;10(2):e24273. doi: 10.1016/j.heliyon.2024.e24273 (PMC10825489; doi:10.1016/j.heliyon.2024.e24273)
Supplement: Multimedia component 1 [file mmc1.docx]

**Table S1.** Survey Instrument

| Code | Items |
| --- | --- |
| HCS1 | I think my health depends on how well I take care of myself. |
| HCS2 | I am actively engaged in the prevention of disease and illness. |
| HCS3 | I think taking preventive measures help to stay healthy. |
| HCS4 | Living a healthy life is important to me. |
| HCS5 | I am constantly examining my health. |
| HMO1 | I usually value my health. |
| HMO2 | I have good knowledge to prevent health issues. |
| HMO3 | I try to prevent health problems before I feel any symptoms. |
| HMO4 | I try to protect myself against health hazards I hear about. |
| HMO5 | I am concerned about health hazards and try to take action to prevent them |
| PIN1 | I would like to explore new consumer goods. |
| PIN2 | I am usually the first to try out new food among my peers. |
| PIN3 | I openly accept new ways of producing food products |
| PIN4 | I like to try new things. |
| PIN5 | I am interested in trying new food products. |
| PCM1 | Most people in my group consumed plant-based meat. |
| PCM2 | Many people with whom I usually communicate consume plant-based meat. |
| PCM3 | Most people in my community consume plant-based meat. |
| PCM4 | I know many people are consuming plant-based meat. |
| PCM5 | Consumption of plant-based meat is getting popular. |
| PCT2 | Prices of plant-based meat is reasonable. |
| PCT4 | I am pleased with the plant-based meat prices. |
| PCT5 | I am satisfied with the price of the plant-based meat. |
| PPV1 | Plant based meat offers good value for money. |
| PPV2 | Plant based meat is beneficial |
| PPV3 | Consumption of plant-based meat is valuable to me. |
| PPV4 | I think consumption of plant-based meat is worthwhile. |
| PPV5 | Overall, using plant-based meat carries good value to me. |
| ICP1 | I propose to use plant-based meat in the future. |
| ICP2 | I will always try to use plant-based meat in daily life in the future. |
| ICP3 | I plan to use plant-based meat frequently to manage my health in the future. |
| ICP4 | I would be willing to develop a habit of using plant-based meat soon. |
| ICP5 | I predict I will use plant-based meat. |
| ACO | How frequently you consume plant-based meat? |

**Note:** HCS - Health Consciousness; HMO - Health Motivation; PIN - Personal Innovativeness; PCM - Perceived Critical Mass; PCT - Perceived Cost; PPV - Perceived Product Value, ICP - Intention to Consume Plant Based Meat; ACO - Actual Consumption of Plant Based Meat
