# Supplementary material for: Modelling the mass consumption potential of Plant Based Meat: Evidence from an emerging economy
Source: Heliyon. 2024 Jan 8;10(2):e24273. doi: 10.1016/j.heliyon.2024.e24273 (PMC10825489; doi:10.1016/j.heliyon.2024.e24273)
Supplement: Multimedia component 2 [file mmc2.docx]

**Table S2.** Loading, Cross-Loading, and Fornell–Larcker Criterion

|  | HCS | HMO | PIN | PCM | PCT | PPV | ICP | ACO |  |
| --- | --- | --- | --- | --- | --- | --- | --- | --- | --- |
| HCS1 | 0.550 | 0.237 | 0.247 | 0.157 | 0.316 | 0.061 | 0.227 | -0.065 |  |
| HCS2 | 0.857 | 0.556 | 0.592 | 0.567 | 0.475 | 0.293 | 0.454 | 0.131 |  |
| HCS3 | 0.824 | 0.507 | 0.536 | 0.517 | 0.412 | 0.287 | 0.417 | 0.121 |  |
| HCS4 | 0.788 | 0.499 | 0.447 | 0.462 | 0.354 | 0.196 | 0.378 | 0.042 |  |
| HCS5 | 0.742 | 0.498 | 0.567 | 0.492 | 0.420 | 0.312 | 0.343 | 0.021 |  |
| HMO1 | 0.302 | 0.535 | 0.308 | 0.140 | 0.056 | 0.143 | 0.089 | 0.026 |  |
| HMO2 | 0.487 | 0.706 | 0.491 | 0.381 | 0.287 | 0.325 | 0.288 | 0.053 |  |
| HMO3 | 0.371 | 0.777 | 0.338 | 0.343 | 0.251 | 0.213 | 0.267 | 0.118 |  |
| HMO4 | 0.422 | 0.791 | 0.419 | 0.369 | 0.268 | 0.270 | 0.245 | 0.083 |  |
| HMO5 | 0.582 | 0.731 | 0.465 | 0.433 | 0.336 | 0.321 | 0.282 | 0.058 |  |
| PIN1 | 0.536 | 0.493 | 0.738 | 0.514 | 0.492 | 0.297 | 0.374 | 0.003 |  |
| PIN2 | 0.600 | 0.506 | 0.862 | 0.615 | 0.476 | 0.387 | 0.457 | 0.106 |  |
| PIN3 | 0.527 | 0.447 | 0.801 | 0.555 | 0.458 | 0.333 | 0.383 | 0.042 |  |
| PIN4 | 0.541 | 0.488 | 0.857 | 0.609 | 0.452 | 0.445 | 0.447 | 0.065 |  |
| PIN5 | 0.321 | 0.313 | 0.656 | 0.472 | 0.342 | 0.441 | 0.376 | 0.079 |  |
| PCM1 | 0.443 | 0.414 | 0.649 | 0.776 | 0.461 | 0.402 | 0.447 | 0.113 |  |
| PCM2 | 0.493 | 0.454 | 0.621 | 0.859 | 0.549 | 0.451 | 0.501 | 0.172 |  |
| PCM3 | 0.457 | 0.394 | 0.503 | 0.824 | 0.465 | 0.412 | 0.422 | 0.067 |  |
| PCM4 | 0.434 | 0.359 | 0.446 | 0.717 | 0.404 | 0.398 | 0.378 | 0.052 |  |
| PCM5 | 0.521 | 0.326 | 0.526 | 0.734 | 0.555 | 0.524 | 0.534 | 0.167 |  |
| PCT2 | 0.393 | 0.275 | 0.465 | 0.487 | 0.817 | 0.422 | 0.533 | 0.148 |  |
| PCT4 | 0.473 | 0.356 | 0.501 | 0.592 | 0.877 | 0.479 | 0.611 | 0.114 |  |
| PCT5 | 0.433 | 0.281 | 0.442 | 0.488 | 0.800 | 0.486 | 0.561 | 0.099 |  |
| PPV1 | 0.255 | 0.280 | 0.365 | 0.481 | 0.422 | 0.800 | 0.430 | 0.171 |  |
| PPV2 | 0.288 | 0.365 | 0.406 | 0.502 | 0.513 | 0.872 | 0.457 | 0.165 |  |
| PPV3 | 0.256 | 0.273 | 0.406 | 0.414 | 0.398 | 0.809 | 0.366 | 0.095 |  |
| PPV4 | 0.210 | 0.225 | 0.286 | 0.296 | 0.367 | 0.652 | 0.281 | 0.067 |  |
| PPV5 | 0.204 | 0.270 | 0.392 | 0.441 | 0.423 | 0.689 | 0.378 | 0.072 |  |
| ICP1 | 0.331 | 0.286 | 0.459 | 0.460 | 0.377 | 0.421 | 0.691 | 0.098 |  |
| ICP2 | 0.348 | 0.236 | 0.350 | 0.419 | 0.572 | 0.383 | 0.815 | 0.185 |  |
| ICP3 | 0.456 | 0.327 | 0.411 | 0.547 | 0.597 | 0.446 | 0.853 | 0.113 |  |
| ICP4 | 0.404 | 0.290 | 0.453 | 0.472 | 0.605 | 0.398 | 0.762 | -0.013 |  |
| ICP5 | 0.217 | 0.092 | 0.202 | 0.190 | 0.263 | 0.123 | 0.506 | -0.025 |  |
| ACO | 0.084 | 0.100 | 0.077 | 0.153 | 0.144 | 0.155 | 0.110 | 1.000 |  |
| *Fornell-Larcker Criterion* | | | | | | | | | |
| HCS | 0.760 |  |  |  |  |  |  |  |  |
| HMO | 0.622 | 0.714 |  |  |  |  |  |  |  |
| PIN | 0.647 | 0.574 | 0.787 |  |  |  |  |  |  |
| PCM | 0.604 | 0.497 | 0.706 | 0.784 |  |  |  |  |  |
| PCT | 0.522 | 0.367 | 0.565 | 0.630 | 0.832 |  |  |  |  |
| PPV | 0.318 | 0.373 | 0.485 | 0.565 | 0.556 | 0.769 |  |  |  |
| ICP | 0.491 | 0.351 | 0.521 | 0.591 | 0.684 | 0.506 | 0.736 |  |  |
| AOC | 0.084 | 0.100 | 0.077 | 0.153 | 0.144 | 0.155 | 0.110 | 1.000 |  |

**Note:** HCS - Health Consciousness; HMO - Health Motivation; PIN - Personal Innovativeness; PCM - Perceived Critical Mass; PCT - Perceived Cost; PPV - Perceived Product Value, ICP - Intention to Consume Plant-Based Meat; ACO - Actual Consumption of Plant Based Meat
